# Changing Characteristics of Pharmaceutical Prices in China Under Centralized Procurement Policy: A Multi-Intervention Interrupted Time Series

**DOI:** 10.3389/fphar.2022.944540

**Published:** 2022-07-15

**Authors:** Hongfei Long, Ying Yang, Xin Geng, Zongfu Mao, Zhenhua Mao

**Affiliations:** ^1^ Dong Fureng Economic and Social Development School, Wuhan University, Wuhan, China; ^2^ Global Health Institute, Wuhan University, Wuhan, China; ^3^ School of Public Health, Wuhan University, Wuhan, China

**Keywords:** centralized procurement, group purchasing, pharmaceutical price, price ratio, China

## Abstract

**Objective**: National centralized drug procurement organized by the Chinese government currently represents the largest group purchasing organization worldwide, to establish a reasonable price formation mechanism. This study aimed to evaluate the effects of centralized procurement policy on drug price and price ratio in China.

**Method**: Monthly drug procurement data of public medical institutions were extracted from the national procurement database, including 11 pilot cities and 36 months from January 2018 to December 2020. Centralized procured INNs (International Nonproprietary Names) (*n* = 25) and their alternative INNs (*n* = 96) were selected as study samples. Centralized procured INNs were divided into bid-winning and non-winning products according to the bidding results. Drug price, price distribution, and price ratio were measured. Multi-intervention interrupted time series analysis was performed to estimate the policy impacts in two centralized procurement periods.

**Results**: The price of centralized procured INNs showed an immediate drop of 44.57% (*β* = -0.59, *p* < 0.001) at the policy implementation, among which bid-winning drugs decreased by 61.71% (*β* = -0.96, *p* < 0.001). No significant change in the price level or trends was found for non-winning products and alternative drugs in the first-year procurement period (all *p*-values > 0.05). During the second-year procurement period, alternative drugs in four therapeutic categories detected significant increases in the price level (all *p*-values < 0.05). The overall coefficient of variation of price distribution exhibited upward trends after policy implementation. Among the most centralized procured INNs, the price ratio between certificated generics (generics that have passed the consistency evaluation) and original drugs declined significantly after policy intervention (*p* < 0.05), whereas the price ratio between uncertificated and certificated generics increased significantly (*p* < 0.05).

**Conclusion**: Chinese government-organized group purchasing resulted in prominent price reduction of bid-winning drugs. The policy observed a short-term “spillover” effect of synergistic price reduction, while the effect wore off after 1-year procurement period. The extremely dispersed price distribution, as well as unreasonable price ratios, requires further effective price regulation means.

## 1 Background

Universal access to affordable medicines and healthcare services despite a consistently surging medicine expenditure remains to be one of the biggest health challenges faced by all countries, and China is no exception. From 2010 to 2018, China spent 30–40% of its total health expenditure on medicines (NHC, 2020), exceeding the figures not only in the United States (12.0%), Japan (18.6%), and Korea but also in the OECD (Organization for Economic Co-operation and Development) countries, average (16.4%) (OECD, 2022). Despite years of radical commitment to a drug price reduction in China, the lack of strong purchasing/negotiation power, integrated pricing strategy, standardized pricing principles, as well as efficient financial incentives have still hindered the establishment of a rational drug-pricing mechanism for the last 2 decades (Hu and Mossialos, 2016; Jiang et al., 2021), which in turn have resulted in skyrocketing medicine expense.

In 2009, China launched its ambitious Universal Health Coverage (UHC) reform to provide all citizens equal access to basic healthcare and medicines. Since then, transparent tendering and pooled procurement have gradually become the major approach to lower drug prices in China (Mao et al., 2014). In 2015, the General Office of the State Council officially called for establishing a regional procurement model to implement centralized procurement of well-competitive essential drugs and generic drugs at the provincial level (General Office of the State Council, 2015). However, due to the significant regional variations in negotiation power, tendering standards, and the legacy of the past “drug mark-up policy”, the early-stage effect of this drug price control action was below expectations (Fu et al., 2015; Zhang et al., 2019).

To improve the overall affordability of quality drugs, promote balanced demand-supply relations, and facilitate healthy competition in the drug market, in November 2018, China launched a novel National Centralized Drug Procurement (NCDP) initiative. The first pilot program of the NCDP was launched in four municipalities (Beijing, Tianjin, Shanghai, Chongqing) and seven subprovincial cities (Shenyang, Dalian, Xiamen, Guangzhou, Shenzhen, Chengdu, and Xi’an) (so-called “4 + 7” procurement). The bidding results revealed that centralized procurement was successful in enhancing payers’ negotiation power to maximize the average bid-winning drug price reduction by 52% (General Office of the State Council, 2019). In January 2021, China further called for the expansion of regular NCDP initiatives and adapt it as a trigger for the establishment of a rational drug-pricing mechanism (General Office of the State Council, 2021). As of February 2022, China has carried out six batches of centralized drug procurement and saved over 260 billion Chinese Yuan (CNY) on medicines for the country. With an average price reduction of 53%, the volume of the bid-winning drugs has accounted for 30% of the annual volume purchased by public medical institutions. (NHSA, 2022).

As the current largest group purchasing organization in the world, certainly, the NCDP initiative in China has generally successfully established a “buyer-cartels” to countervail pharmaceutical companies for quality drugs at lower prices (Huff-Rousselle, 2012; Xing et al., 2022). However, the policy impact on drug price change patterns and mechanisms across different countries/regions may be associated with various contextual factors (e.g., policy environment, healthcare conditions, market maturity, competitiveness, etc) and structural factors (e.g., strategy design, stakeholder dynamics, operating mechanism, affected product categories, etc). Aiming to obtain further empirical insights on the practices and impacts of NCDP policy in China, this exploratory study mainly focuses on examining the mid- to long-term price change and price relation characteristics among different categories of pharmaceutical products under centralized procurement policy.

## 2 Literature Review

### 2.1 Price Change

Fostering the formation of buyer monopoly through integrating purchasing power for price negotiation with seller is known as the theoretical base for the price-cutting outcome brought by centralized procurement (Huff-Rousselle, 2012). Many studies have provided evidence for such price-changing effects (Kim and Skordis-Worrall, 2017; Toulemon, 2018; Dubois et al., 2021). Dubois et al. (2021) found that centralized procurement contributed at least 15% of the drug price reductions among seven low- and middle-income countries between 2015 and 2017.

In addition, our literature review shows that price change characteristics may be associated with multiple barriers and facilitators among different pharmaceutical categories under the centralized procurement policy. Dubois et al. (2021) argued that the impact of centralized procurement mechanisms on product prices was related to the degree of market concentration or level of competitiveness, in that the higher the market concentration of products, the smaller the impact brought by the policy. Besides, the group purchase of innovative high-cost drugs in France revealed that the policy has a greater impact on oligopolistic drug prices than on monopoly prices (Toulemon, 2018). Pérez et al. (2019) mentioned that pharmaceutical enterprises might rapidly raise the product prices 1 or 2 years after initial price reduction, suggesting a need to observe the long-term effects of such policies on drug prices.

In China, Zhang et al. (2019) conducted a systematic review on the impact of the centralized drug procurement policy, in which 29% of the included studies were positive that the policy has facilitated drug price cuts and 13.5% believed that it accelerated the progress of universal access to essential medicines. Huang et al. (2019) analyzed the impact of centralized procurement policy on drug prices of ten “4 + 7” centralized procured drugs. It was found that the price ratio of the bid-winning products compared with the international reference price (3.65) was far lower than that of the non-winning products (7.42), indicating that the price of the bid-winning product gradually cut to a rational level. Chen et al. (2020) and Wang et al. (2021) suggested that in the short run (10 months after the execution of the NCDP pilot), the marked decline in the prices of bid-winning products might have potentially driven the price cut of the non-winning products. Wang et al. (2021) found that despite that the price index for alternative medicines (i.e., drug substances that have an alternative relationship with centralized procured drugs) did not change significantly at the beginning of policy implementation, but gradually a downward trend emerged.

However, there is inadequate evidence regarding the mid- or long-term policy effect on the drug price cut in the context of centralized drug procurement policy in China. Without first deeply understanding the complex factors and triggers of drug price change (Sun et al., 2022), the country may continue to struggle with significant issues such as drug price deviation, drug rebates, inflated prices, and unstandardized procurement practice (Zhang et al., 2019). It is necessary for a more in-depth sub-group analysis to comprehensively reveal the pivotal and regularity of drug price change associated with centralized drug procurement policy in China.

### 2.2 Price Relations

Establishing a rational drug pricing mechanism is the fundamental means and end for the vigorous advocating of the centralized drug procurement initiative in China (Wang, 2020). Therefore, it is of great pragmatic significance to explore the impact of centralized procurement policy on drug price mechanism and price relation (price comparison relationship) in China. Drug price relation/ratio refers to the proportional relationship among prices of different pharmaceutical products in the same market within a certain period, which is known to be one key ingredient for the structure and process of drug price formation (Lu, 2018). Relevant studies on drug price relations have revealed the significance of price differences, price comparisons, and referencing prices in the establishment of a drug price mechanism (Danzon and Chao, 2000; Vieira and Zucchi, 2006; Puig-Junoy and Moreno-Torres, 2010; Dylst and Simoens, 2011).

In China, relevant research on price gaps between generic and original drugs presents the dilemma in the current drug pricing mechanism—the inconsistent quality and efficacy of domestic generic drugs compared with originators, thus hindering the effective formation of market competitiveness of generic drugs (Zhang and Hu, 2016). Efforts have been made since 2012, the Generic Consistency Evaluation (GCE) work was conducted and promoted by the National Medical Products Administration (NMPA) to ensure that marketed generic drugs became bioequivalent to their corresponding original brand-name drugs and has made great strides (State Council Information Office, 2019). Generic drugs are certified for quality and efficacy consistency through pharmacokinetics equivalence and bioequivalence trials (therapeutic equivalence trials are exempted) (Hu, 2021). Although whereafter the National Healthcare Security Administration (NHSA) reports that certificated generics are equivalent to original drugs in both clinical therapeutic efficacy and safety (i.e., therapeutic equivalence) (CCTV, 2021), nor does it seem to completely reassure prescribers and patients (He et al., 2021; Qu et al., 2021). The existence of bioequivalence with originators can theoretically improve the market competitiveness of generic drugs and promote overall price reduction, while it is not the case in China (Chen et al., 2018; Rong and Sun, 2018; Oncu et al., 2020). Li et al. (2012) reported that the price of original drugs in the selected province was 3.6 times that of generic drugs. In 2014, Zhang et al. (2016) found in a drug market survey in Shanghai that the overall price ratio of generic drugs to original drugs was 0.54, indicating the latter was about 1.85 times more expensive than that of the former. An investigation of the prices of 27 commonly used drugs in 31 provinces (Rong and Sun, 2018) found that not only the prices of both original and generic drugs in China were significantly higher than international reference prices, but also the price levels of certain generic drugs drastically varied across provinces in their drug price distribution analysis. The high price difference between generics and originators, as well as the low market share of generics were blamed to the motives of personal interests which drives physicians to prescribe expensive drugs where prices had not been cut off, thus consistency evaluation alone had not generally promoted the substitution of generics.

Recent NCDP policy adopts the GCE as the gate-keeper for qualified generic drugs to participate in the price competition with original drugs in state-wide centralized bidding. All the drugs involved in the market under centralized procurement are comparable as the policy requires that their quality and efficacy are consistent. This provides a pragmatic premise for measuring drug price relation changes. However, to the best of our knowledge, there is limited research focusing on the changes in product price differences and price relations associated with the implementation of the national centralized procurement policy in China.

In light of the abovementioned evidence and current research gaps, this study aims to apply the large-scale NCDP program data to 1) explore the direct and “spillover” effects of NCDP policy on the price-changing patterns and characteristics among the bid-winning drugs, non-winning drugs, and alternative drugs in both short- and long-term, and 2) estimate the effects of the NCDP policy on the change in drug price distribution and price relation among different pharmaceutical categories.

## 3 Methods

### 3.1 Description of the Policy Intervention

As a pharmaceutical reform with multi-dimensional target attributes and multiple intervention measures, the NCDP policy has been systematically elaborated on its policy practices by previous scholars (Chang, 2021; Hu, 2021; Yuan et al., 2021). In this study, we focus on the policy measures mostly directly related to drug price changes. As is well-known, improving seller’s competition is an effective approach to price reduction. In the national centralized drug procurement, the Chinese government adopted the following measures to improve competition: 1) Enhancing competition between quality-assured generics and originators drugs. Only generic drugs certified for quality and efficacy consistency by NMPA (called certificated generic drugs) as well as corresponding originators are eligible to participate in NCDP. They are considered of equal quality and efficacy, and thus were assigned as the same quality level for price competition during centralized bidding (General Office of the State Council, 2019). 2) Merging dosage form and specification. The dosage forms and specifications of drugs were properly merged, and some products with “strange” specifications and dosage forms were excluded from the centralized bidding. Price competition was conducted in the unit of generic name. 3) The limited number of bid-winning enterprises. To improve the intensity of bidding, only the lowest bidder wins the bid in the first bidding of “4 + 7” pilot, and the number of bid-winner adjusted to one to three in the second bidding.

Compared with the first bidding, the core purpose of the second renewal bidding is to achieve “three stabilization” (market expectation, price level, and clinical medication) (NHSA, 2021), rather than a further significant decline in drug price. Therefore, unlike the first bidding, the organization and decision-making of the second renewal bidding were delegated from the state to pilot cities. The bid-winning enterprises of each INN in the two biddings are also different, see Supplementary Table S1.

In the present study, the implementation of centralized bidding results was assigned as the intervention to quantify the policy impact on drug prices. In March 2019, all public medical institutions in “4 + 7” pilot cities started carrying out the bid-winning results of the first bidding. After the end of the first 1-year procurement period, second renewal bidding was conducted and the bidding results were implemented in April 2020. Thus, the observation months (January 2018 to December 2020) were divided into three periods: 1) pre-intervention period (January 2018 to February 2019), 2) the first procurement period (March 2019 to March 2020), and 3) second procurement period (April to December 2020).

### 3.2 Data Sources and Samples

The data used in this study were extracted from the China Drug Supply Information Platform (CDSIP), which summarizes and maintains the drug procurement data of 31 provincial drug bidding and procurement platforms in mainland China. Procurement data extracted from CDSIP include drug name (International Nonproprietary Name, INN), drug code, the name of the medical institution, procurement date, dosage form, specification, packaging, manufacturer, unit price, procurement unit, procurement quantity, procurement expenditures, etc. The integrality and accuracy of CDSIP procurement data are largely endorsed, in that the Chinese government mandates all public medical institutions shall purchase the drugs to be used through the government-led provincial drug centralized procurement platform (General Office of the State Council, 2015). By the end of 2020, CDSIP data covered 48,205 public medical institutions in 31 provinces, including 9176 public hospitals and 39,029 public healthcare centers (Yang et al., 2022). Despite its full coverage of public medical institutions, the CDSIP is estimated to cover over 80% of drug purchasing data from national health facilities in mainland China. The procurement data from the private departments may not be included.

In this study, drug procurement data of all public medical institutions from all eleven “4 + 7” pilot cities were extracted for analysis. The scope of medicines includes centralized procured drugs and their alternative drugs, which were defined as follows:

1) Centralized procured drug refers to the INNs covered by the centralized procurement catalog, which was announced by the Joint Procurement Office (JPO) in the tender document (Joint Procurement Office, 2018a). A total of 25 INNs were procured during the “4 + 7” pilot and thus were included and defined as centralized procured INNs in this study. Furthermore, centralized procured INNs we dichotomized into bid-winning and bid-non-winning drugs according to the bidding results (Joint Procurement Office, 2018b). Drug products that won the bid in the JPO-organized centralized bidding were identified as bid-winning drugs, otherwise were bid-non-winning drugs. We also sorted centralized procured INNs into off-patent original branded products (i.e., original drugs) and generic products based on the *Catalogue of Marketed Drug in China* (NMPA, 2017). Generic drugs were further distinguished by whether had passed the GCE as of March 2019.
2) Alternative drugs were defined as the clinically substitutable drugs of the same therapeutic category with centralized procured drugs, which were not covered by the “4 + 7” centralized procurement catalog. We included alternative drugs following the list of “alternative drugs” provided by the NHSA in the *Monitoring Plan for Centralized Drug Procurement and Use Pilot Work* (NHSA, 2019a). Among them, several drugs were procured in the centralized procurement work of subsequent batches during our observation period, and thus were excluded from our sample. A total of 96 alternative drugs were included in the analysis.

Data were managed using ATC (Anatomical Therapeutic and Chemical) code. Included drugs were sorted into 12 therapeutic categories based on the ATC 2-level code: hypotensive drugs (C08/09), lipid-modifying agents (C10), antiepileptics (N03), psycholeptics (N05), psychoanaleptics (N06), antineoplastic agents (L01), antibacterials for systemic use (J01), antivirals for systemic use (J05), antidiarrheals (A07), antithrombotic agents (B01), antiinflammatory and antirheumatic products (M01), and drugs for obstructive airway diseases (R03). The detailed information on included drugs is listed in Supplementary Table S2.

### 3.3 Definition of Outcome Variables

#### 3.3.1 Measure of Drug Price

The unit price of each procured drug was calculated based on its defined daily dosage (DDD) defined by the World Health Organization (WHO) (WHO Collaborating Centre for Drug Statistics Methodology, 2018) in absolute monetary terms (CNY). The calculation of drug price is as follows:

$$Y\;=\overset{n}{\underset{i=1}{\sum }}C_{i}/\overset{n}{\underset{i=1}{\sum }}\left(\frac{U_{i}P_{i}}{DDD_{i}}\times N_{i}\right)$$ (1)

where *Y* refers to unit price, *C*_*i*_ represents the cumulative procurement costs of drug product *i*, *DDD*_*i*_ refers to the DDD value of product *i*; *U*_*i*_ refers to the unit ingredient of product *i*; *P*_*i*_ refers to the packing specification of product *i*; *N*_*i*_ refers to the number of product *i*.

#### 3.3.2 Measure of Price Distribution

In the present study, we described the price distribution among different drug products within the same therapeutic category. The indicators of dispersion tendency were employed including median, range, coefficient of variation (CV), etc.

#### 3.3.3 Measure of Price Ratio

The price relation of drugs can be measured by the price ratio (PR) of different products. Following the previous studies’ approach (Vieira and Zucchi, 2006; Zhang et al., 2016; Zhang and Hu, 2016), we first calculated the price ratio between generic drugs and original drugs: PR_1_ = price of generic drug/price of the original drug. The difference in quality and efficacy between generic drugs and original drugs would lead to the lack of practical significance of their price comparison (Zhang et al., 2016). Therefore, we further calculated the price ratio between certificated generic drugs and original drugs: PR_2_ = price of certificated generic drug/price of original drug, as well as the price ratio between uncertificated generic drugs and certificated generic drugs: PR_3_ = price of uncertificated generic drug/price of certificated generic drug.

### 3.4 Statistical Analysis

The descriptive statistical method was first employed. To visualize the policies’ effects, we graphed monthly trends in drug price of each drug category, fitting the curves of monthly moving averages. Indicators of dispersion tendency (median, range, and CV), as well as violin plots, were applied to describe the price distribution characteristics of different products.

This study applied multi-intervention interrupted time series (ITS) analysis (Ariel, 2015) to estimate the effect of centralized procurement policy in two bidding periods. The regression model was constructed as follows:

$$\begin{array}{c} Y_{it}=\alpha +\beta_{0}*time+\beta_{1}*intervention_{1}+\beta_{2}*time*intervention_{1}+\beta_{3}*intervention_{2}\\ +\beta_{4}*time*intervention_{2}+\mu_{it}+\varepsilon_{it} \end{array}$$ (2)

where *Y*_*it*_ refers to outcome variables, i.e. drug price or price ratio. *time* is a continuous variable of observation months ranging from 1 to 36. *intervention*_*1*_ and *intervention*_*2*_ are dummy variables of policy intervention time. *intervention*_*1*_ was coded 1 in the first bidding period (March 2019 to March 2020), otherwise coded 0. *intervention*_*2*_ was coded 1 in the second bidding period (April to December 2020), otherwise coded 0. *μ*_*it*_ is the fixed effect of drug products. *ε*_*it*_ refers to the random error term. *α* and *β* estimated the intercept and slope of drug price in the pre-intervention period of centralized procurement policy, respectively. *β*_*1*_ and *β*_*3*_ estimated the immediate level change of dependent variables at the point of implementing the first and second bidding results, respectively. *β*_*2*_ and *β*_*4*_ estimated the slope change of dependent variables during the first and second procurement periods. Data were managed and analyzed in Stata version 15.0 (Stata Corp LP, College Station, TX, United States). A difference with *p* < 0.05 was considered to indicate statistical significance.

## 4 Results

### 4.1 Bid-Winning Prices in Centralized Bidding

In the pre-intervention period, the median price of 25 centralized procured INNs was 7.33 CNY. After centralized bidding, the median price of bid-winning products is 1.65 CNY (first bidding) and 1.26 CNY (second bidding), respectively. The price decline of first bidding compared with the pre-intervention period ranges from 2.16 to 94.65%, with an average of 63.16%. The price decline of second bidding compared with the pre-intervention period ranges from 31.87 to 97.71%, with an average of 68.86%.

**FIGURE 1:**
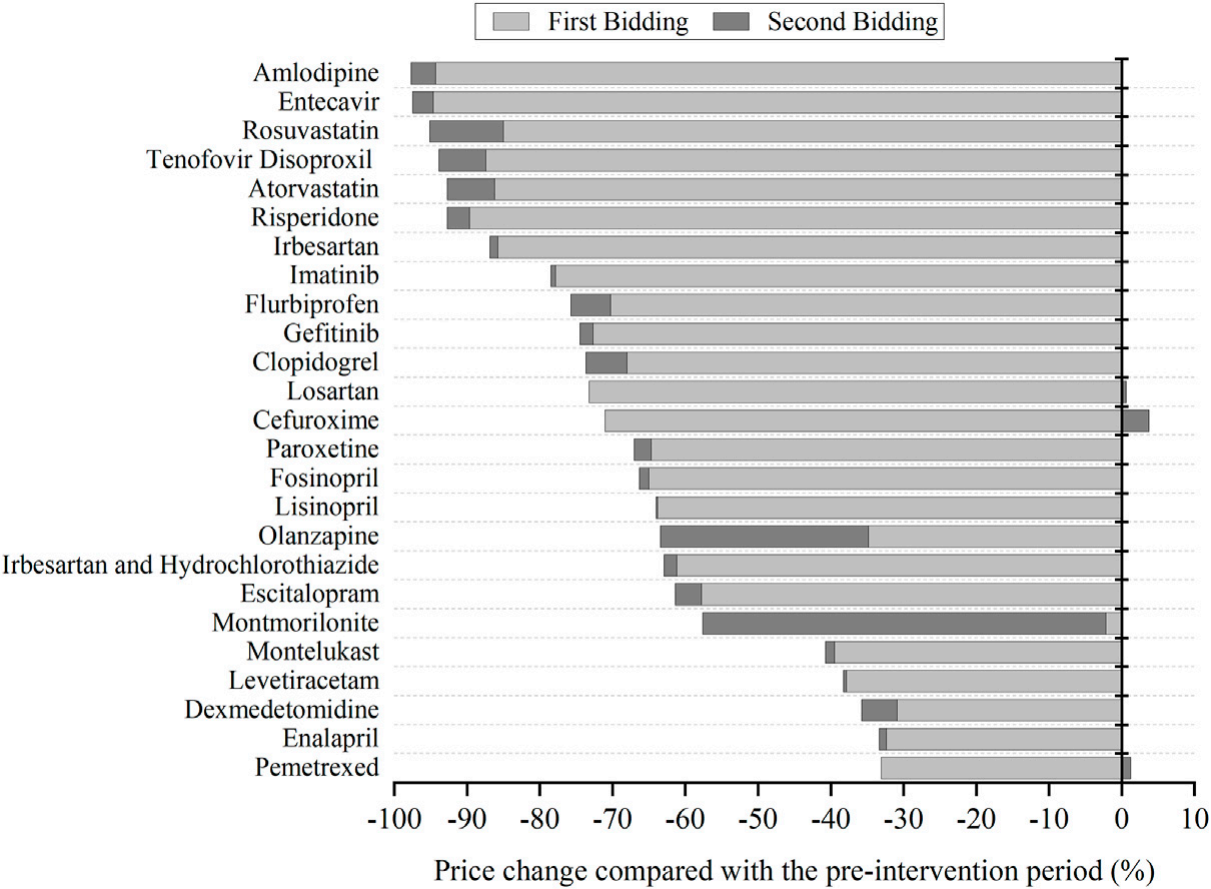
The change of bid-winning prices for each centralized procured INN in two centralized biddings compared with the pre-intervention period.

Figure 1 presents the price changes of bid-winning drugs in two centralized biddings. For most INNs, the prominent price decline occurred in the first bidding period, and then observed a slight decrease in the second bidding. For several INNs, such as Olanzapine and Montmorillonite, a significantly prominent price reduction was also observed in second bidding. Three INNs (Losartan, Cefuroxime, and Pemetrexed) observed price raising in the second bidding against the first bidding.

### 4.2 Price Change Under Policy Impact

#### 4.2.1 Price Change of Bid-Winning and Bid-non-winning Products

**FIGURE 2:**
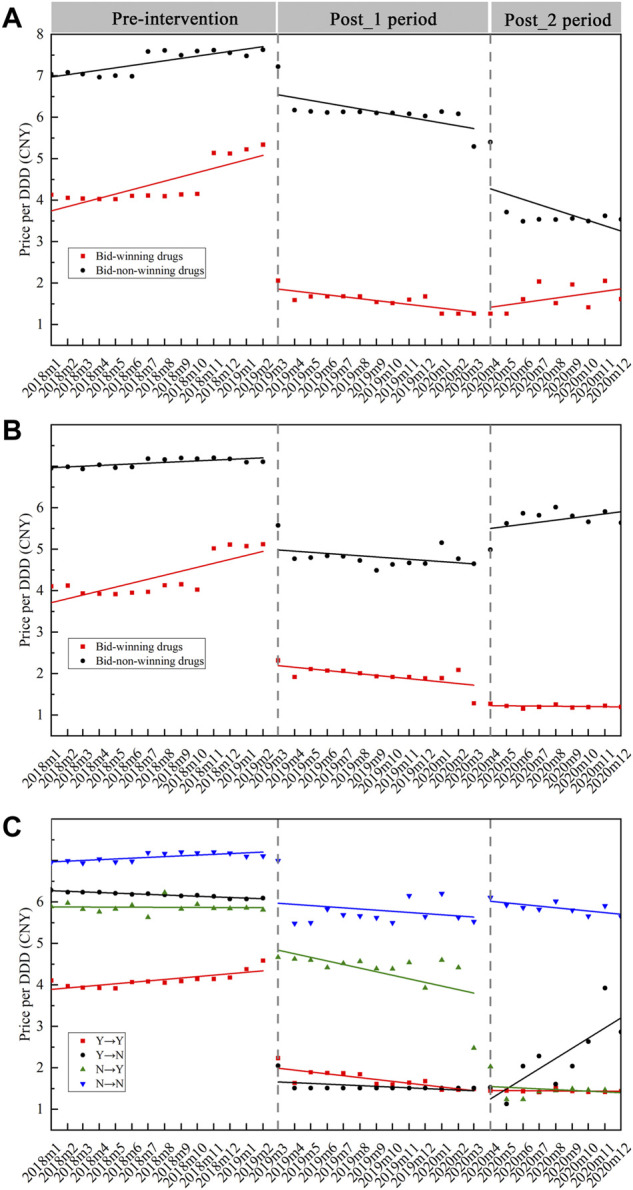
Monthly trends in the price of bid-winning and bid-non-winning products. **(A)** Bid-winning and bid-non-winning products in the first procurement period, **(B)** bid-winning and bid-non-winning products in the second procurement period, **(C)** the cross-bidding results among two procurement periods. *Note.* DDD, defined daily dose; CNY, Chinese yuan.

Centralized procured INNs were divided into bid-winning and bid-non-winning products. Moreover, according to the discrepant bid-winning results in two centralized biddings, they were sorted into four categories: 1) products that won the bid in two centralized biddings, coded as Y→Y; 2) products that won the first bid but failed in the second bidding, coded as Y→N; 3) products failed in the first bidding and won the second bid, coded as N→Y; and 4) products that did not win the bid in two centralized biddings, coded as N→N. Figure 2 graphs the monthly price trends of products in different bidding results between January 2018 and December 2020. The corresponding multi-intervention ITS results are presented in Table 1. In two procurement periods, the price level of bid-winning drugs significantly decreased by 61.71% (e^−0.96^–1) (*β* = −0.96, *p* < 0.001) after first bidding and 23.97% (e^−0.27^–1) (*β* = −0.27, *p* < 0.001) after second bidding, respectively. The price change of bid-non-winning drugs had no significance (all *p*-values > 0.05) in two procurement periods (Figures 2A,B).

**TABLE 1:** Multi-intervention ITS quantifying the impact of centralized procurement policy on prices of bid-winning and bid-non-winning products.

Categories	Involved INNs	Level_1		Trend_1		Level_2		Trend_2	
		*β*_*1*_	*p*	*β*_*2*_	*p*	*β*_*3*_	*p*	*β*_*4*_	*p*
First procurement period									
Bid-winning	25	−0.96	**0.000**	−0.01	0.374	−0.10	0.081	0.02	0.061
Bid-non-winning	25	−0.01	0.839	−0.01	0.230	−0.20	**0.000**	−0.001	0.952
Second procurement period									
Bid-winning	25	−0.76	**0.000**	−0.01	0.201	−0.27	**0.000**	0.01	0.321
Bid-non-winning	23	−0.12	**0.001**	−0.003	0.461	0.06	0.145	0.01	0.187
Cross-bidding results^a^									
Y→Y	22	−0.90	**0.000**	−0.01	0.334	−0.11	**0.023**	0.01	0.464
Y→N	4	−1.56	**0.000**	−0.01	0.727	0.05	0.827	0.14	**0.002**
N→Y	17	−0.15	**0.016**	−0.03	**0.001**	−0.53	**0.000**	0.01	0.653
N→N	23	−0.04	0.109	−0.003	0.385	0.01	0.730	0.001	0.798

a Y→Y represents products that won the bid in two centralized biddings, Y→N represents products that won the first bid but failed in the second bidding; N→Y represents products that failed in the second bidding and won the second bid, and N→N represents products that did not win the bid in two centralized biddings.

INNs, International Nonproprietary Names. Bold values indicate regression coefficients with statistical significance (*p* < 0.05). Fixed effect of drug products was applied.

For Y→Y products, the immediate price decline of 59.30% (*β* = −0.90, *p* < 0.001) and 10.51% (*β* = −0.11, *p* = 0.023) were found at the start of the first and second procurement period, respectively; the change in price slopes during two procurement periods showed no significance (all *p*-values > 0.05). As for Y→N products, an immediate price decline of 78.92% (*β* = −1.56, *p* < 0.001) was found after first bidding; the price level had no significant change after second bidding (*p* > 0.05) and showed an increasing trend during the second procurement period (*β* = 0.16, *p* = 0.002, 14.80% increment). With regards to N→Y products, the significant decrease in price level change (*β* = −0.15, *p* < 0.001, 13.93% decrease) and trend change (*β* = −0.03, *p* = 0.001, 2.76% decrease) were detected in the first procurement period; the price significantly decreased by 41.14% (*β* = -0.53, *p* < 0.001) at the start of second procurement period. For N→N products, the increase in price level and trend showed no significance (all *p*-values > 0.05) in the first procurement period; the decrease in price level and trend also had no significance (all *p*-values > 0.05) in the second procurement period (Figure 2C).

#### 4.2.2 Price Change of Centralized Procured and Alternative Drugs by Therapeutic Category

**FIGURE 3:**
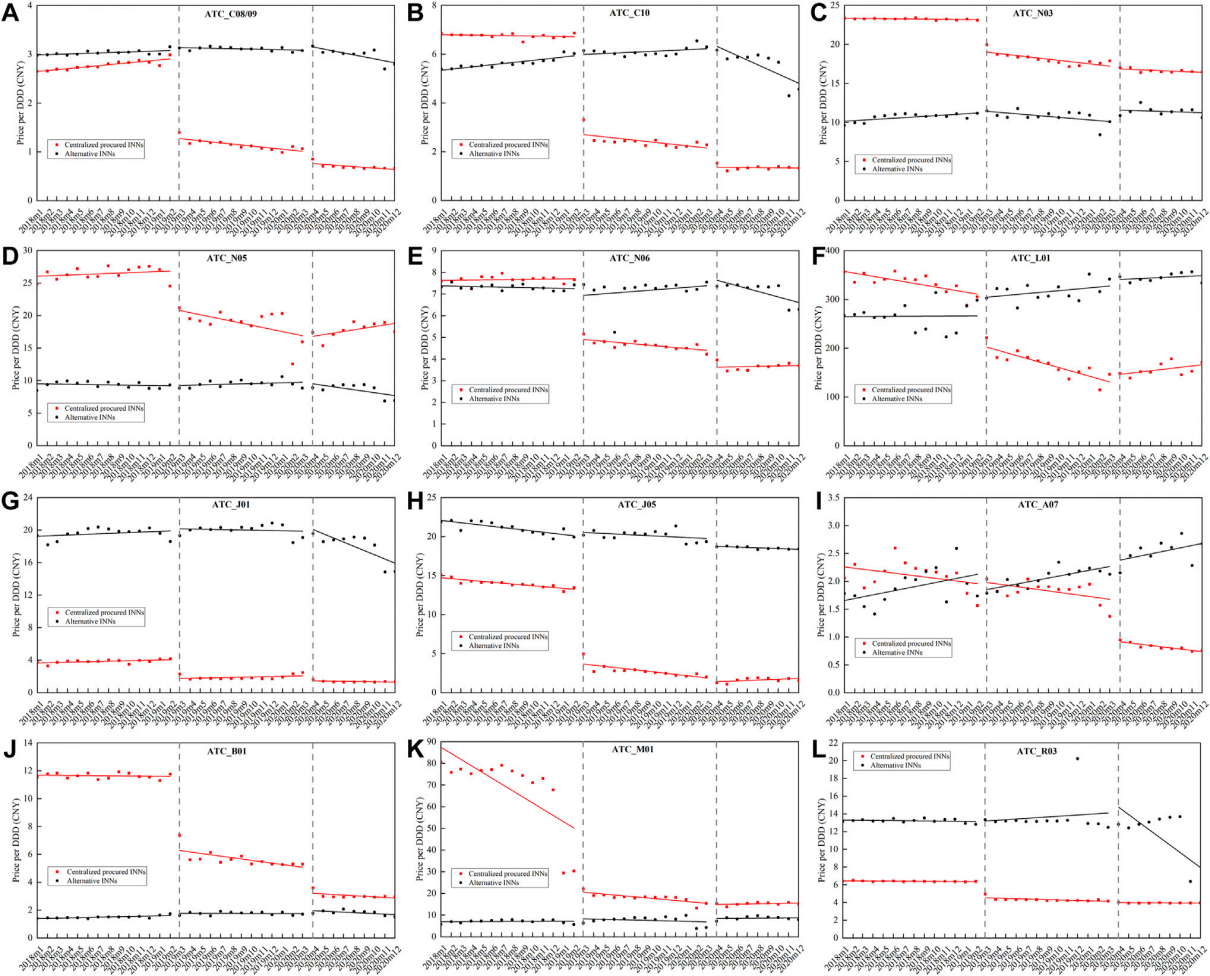
Monthly trends in the price of centralized procured drugs and alternative drugs, stratified by therapeutic categories. **(A)** Hypotensive drugs (C08/09), **(B)** lipid-modifying agents (C10), **(C)** antiepileptics (N03), **(D)** psycholeptics (N05), **(E)** psychoanaleptics (N06), **(F)** antineoplastic agents (L01), **(G)** antibacterials for systemic use (J01), **(H)** antivirals for systemic use (J05), **(I)** antidiarrheals (A07), **(J)** antithrombotic agents (B01), **(K)** antiinflammatory and antirheumatic products (M01), **(L)** drugs for obstructive airway diseases (R03). *Note*: ATC, Anatomical Therapeutic and Chemical.

Figure 3 outlines the monthly price change of centralized procured INNs and alternative INNs from January 2018 to December 2020, stratified by therapeutic categories. The corresponding multi-intervention ITS results are summarized in Table 2. The overall price of centralized procured drugs significantly decreased by 44.57% (*β* = −0.59, *p* < 0.001) and 19.27% (*β* = −0.21, *p* = 0.001) at the start of the first and second procurement period, respectively. The trend change in the overall price of centralized procured drugs showed no significance during two procurement periods (all *p*-values > 0.05). The level and trend change in the overall price of alternative drugs had no significance in the first procurement period (all *p*-values > 0.05). The overall price of alternative drugs showed no significant change in the level and slope in the first procurement period (all *p*-values > 0.05), whereas the price level significantly increased by 7.04% (*β* = 0.07, *p* = 0.036) and decreased by 1.59% (*β* = -0.02, *p* = 0.018) in price slope in the second procurement period.

**TABLE 2:** Multi-intervention ITS quantifying the impact of centralized procurement policy on prices of centralized procured INNs and alternative INNs, stratified by therapeutic categories.

Therapeutic Categories^a^	Centralized procured drugs									Alternative drugs								
	Number of INNs	Level_1		Trend_1		Level_2		Trend_2		Number of INNs	Level_1		Trend_1		Level_2		Trend_2	
		*β*_*1*_	*p*	*β*_*2*_	*p*	*β*_*3*_	*p*	*β*_*4*_	*p*		*β*_*1*_	*p*	*β*_*2*_	*p*	*β*_*3*_	*p*	*β*_*4*_	*p*
Overall^b^	25	−0.59	**0.000**	−0.01	0.182	−0.21	**0.001**	0.02	0.186	96	0.02	0.243	−0.002	0.418	0.07	**0.036**	−0.02	**0.018**
C08/09	7	−0.81	**0.000**	−0.03	**0.000**	−0.27	**0.000**	−0.003	0.790	25	0.02	0.064	−0.004	**0.004**	0.04	**0.010**	−0.01	**0.002**
C10	2	−0.89	**0.000**	−0.02	**0.039**	−0.46	**0.000**	0.02	0.243	8	0.01	0.670	−0.01	**0.035**	0.06	0.131	−0.04	**0.000**
N03	1	−0.19	**0.000**	−0.01	**0.001**	−0.02	0.276	0.01	0.058	7	0.03	0.370	−0.02	**0.003**	0.14	**0.027**	0.01	0.452
N05	3	−0.23	**0.000**	−0.02	**0.043**	−0.02	0.828	0.03	**0.008**	16	0.001	0.983	0.01	0.257	0.01	0.838	−0.03	**0.016**
N06	2	−0.44	**0.000**	−0.01	**0.002**	−0.20	**0.000**	0.01	0.122	10	−0.05	0.397	0.01	0.223	0.06	0.058	−0.02	**0.004**
L01	3	−0.38	**0.000**	−0.03	**0.002**	0.08	0.234	0.05	**0.000**	6	0.13	0.052	0.01	0.416	0.04	0.247	−0.003	0.545
J01	1	−0.85	**0.000**	0.01	0.709	−0.33	**0.001**	−0.03	0.079	6	0.01	0.603	−0.004	0.340	0.05	0.255	−0.03	**0.001**
J05	2	−1.22	**0.000**	−0.04	**0.001**	−0.40	**0.003**	0.09	**0.000**	3	0.02	0.163	0.004	0.144	−0.05	**0.010**	0.001	0.729
A07	1	0.03	0.688	−0.003	0.820	−0.58	**0.000**	−0.01	0.205	4	−0.16	0.075	−0.003	0.809	0.03	0.646	−0.002	0.863
B01	1	−0.60	**0.000**	−0.02	**0.012**	−0.45	**0.000**	0.004	0.754	4	0.09	0.060	−0.01	**0.044**	0.14	**0.041**	−0.02	0.088
M01	1	−0.85	**0.000**	0.02	0.263	−0.05	0.407	0.03	**0.000**	4	0.18	0.275	−0.02	0.458	0.21	0.349	0.02	0.418
R03	1	−0.33	**0.000**	−0.01	0.062	−0.04	**0.034**	0.01	0.076	3	−0.002	0.939	0.01	0.477	0.23	0.323	−0.10	0.063

a Therapeutic category was classified based on the ATC, 2-level code, including hypotensive drugs (C08/09), lipid-modifying agents (C10), antiepileptics (N03), psycholeptics (N05), psychoanaleptics (N06), antineoplastic agents (L01), antibacterials for systemic use (J01), antivirals for systemic use (J05), antidiarrheals (A07), antithrombotic agents (B01), antiinflammatory and antirheumatic products (M01), drugs for obstructive airway diseases (R03).

b The regression model for overall price change applied fixed effect of therapeutic category.

INNs, International Nonproprietary Names. Bold values indicate regression coefficients with statistical significance (*p* < 0.05).

Among 12 therapeutic categories of centralized procured drugs, except for A07 (Montmorillonite) (*p* = 0.688), the immediate decline was detected in the price of the other 11 categories at the start of the first procurement period (all *p*-values < 0.001), with the decline ranging between 17.22% (N03) and 70.54% (J05). During the implementation of first bidding, the trend change significantly decreased in the price of eight therapeutic categories (C08/09, C10, N03, n05, N06, L01, J05, and B01) (all *p*-values < 0.05). At the start of the second procurement period, a significant decline in the price of eight categories (C08/09, C10, N06, J01, J05, A07, B01, and R03) was found (all *p*-values < 0.05). Four (N05, L01, J05, and M01) of the 12 therapeutic categories showed significant increments in price trends during the second procurement period (all *p*-values < 0.05).

In regards to alternative drugs, the immediate price change in all 12 therapeutic categories had no significance (all *p*-values > 0.05) at the start of first procurement period, while the price slope of four categories (C08/09, C10, N03, and B01) decreased significantly (all *p*-values < 0.05). At the implementation of second bidding, the immediate price increases were observed in C08/09 (*β* = 0.04, *p* = 0.010), N03 (*β* = 0.14, *p* = 0.027), and B01 (*β* = 0.14, *p* = 0.041), with the estimated increment of 3.77, 15.14, and 14.91%. The price slope of five categories (C08/09, C10, N05, N06, and J01) increased prominently during the second procurement (all *p*-values < 0.05).

### 4.3 Change of Price Distribution

#### 4.3.1 Centralized Procured Drugs

**FIGURE 4:**
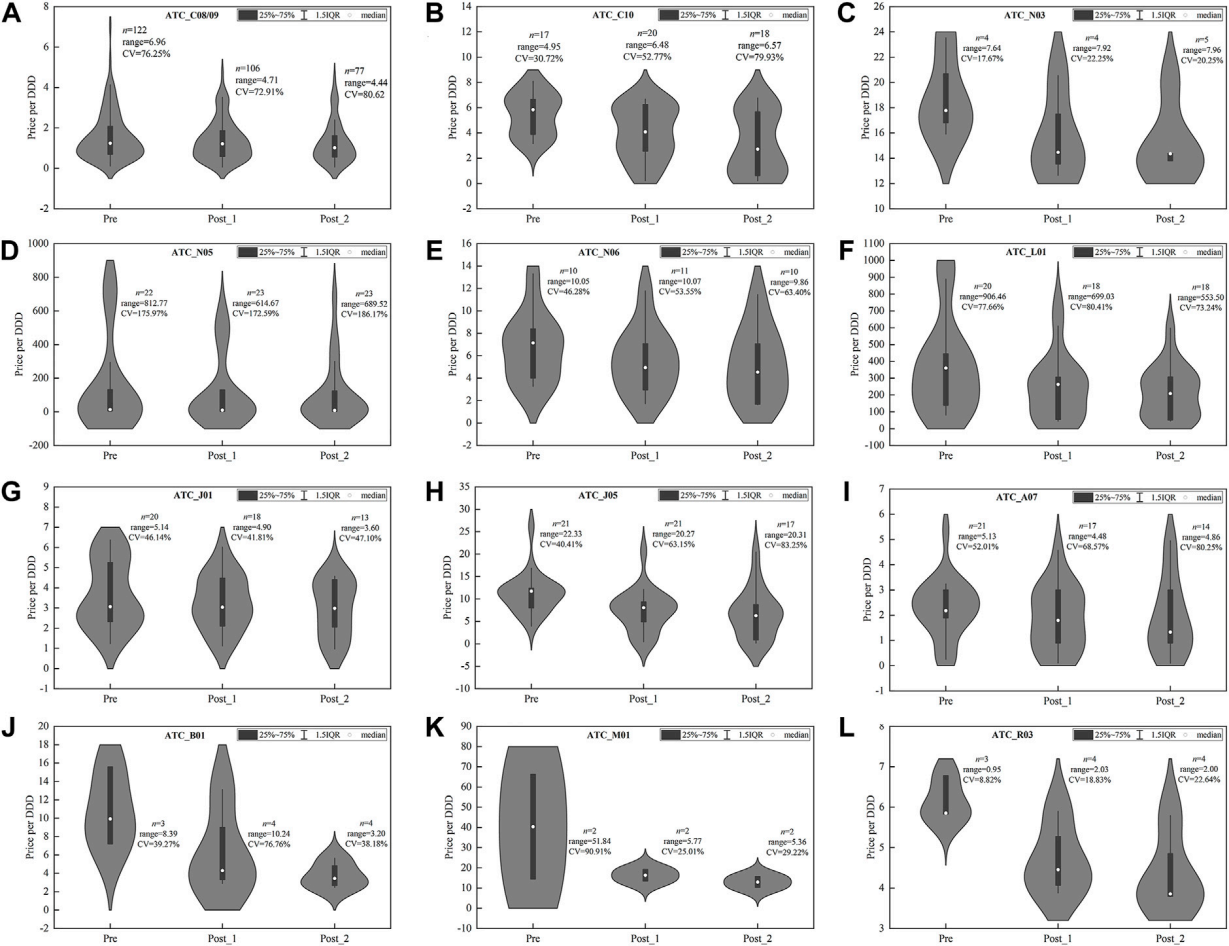
Price distribution of drug products in centralized procured INNs among three observation periods. **(A)** Hypotensive drugs (C08/09), **(B)** lipid-modifying agents (C10), **(C)** antiepileptics (N03), **(D)** psycholeptics (N05), **(E)** psychoanaleptics (N06), **(F)** antineoplastic agents (L01), **(G)** antibacterials for systemic use (J01), **(H)** antivirals for systemic use (J05), **(I)** antidiarrheals (A07), **(J)** antithrombotic agents (B01), **(K)** antiinflammatory and antirheumatic products (M01), **(L)** drugs for obstructive airway diseases (R03). *Note*: DDD, defined daily dose; CV, coefficient of variation.

Figure 4 presents the price distribution of centralized procured INNs among different products in three observation periods, and obvious changes in price distribution were observed after policy intervention.

For some categories, such as C08/09 (Figure 4A), J05 (Figure 4H), B01 (Figure 4J), and M01 (Figure 4K), the range of products’ price distribution decreased markedly in post-intervention periods. In most categories, such as C10 (Figure 4B), N03 (Figure 4C), N06 (Figure 4E), L01 (Figure 4F), J05 (Figure 4H), A07 (Figure 4I), B01 (Figure 4J), M01 (Figure 4K), R03 (Figure 4L), the median and mean of price distribution decreased after policy intervention, and the distribution density moved downward. In several categories, such as C10 (Figure 4B), J05 (Figure 4H), A07 (Figure 4I), and R03 (Figure 4L), the CV of price distribution increased compared with the pre-intervention period. Overall, the medians of CV among the 12 categories were 58.51, 62.38, and 67.02% in three observation periods, respectively, indicating the ascending of dispersion degree.

#### 4.3.2 Alternative Drugs

**FIGURE 5:**
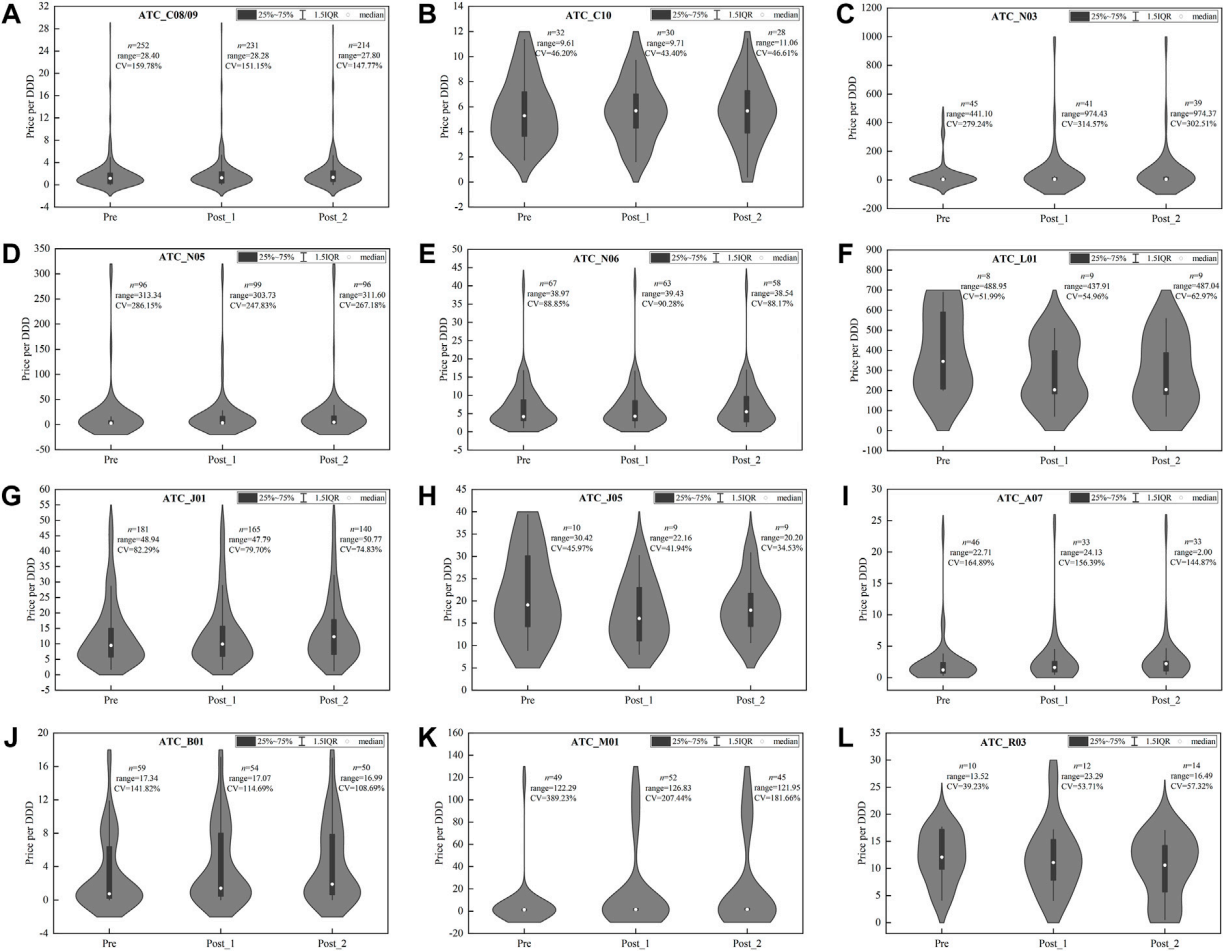
Price distribution of drug products in alternative INNs among three observation periods. **(A)** Hypotensive drugs (C08/09), **(B)** lipid-modifying agents (C10), **(C)** antiepileptics (N03), **(D)** psycholeptics (N05), **(E)** psychoanaleptics (N06), **(F)** antineoplastic agents (L01), **(G)** antibacterials for systemic use (J01), **(H)** antivirals for systemic use (J05), **(I)** antidiarrheals (A07), **(J)** antithrombotic agents (B01), **(K)** antiinflammatory and antirheumatic products (M01), **(L)** drugs for obstructive airway diseases (R03). *Note*: DDD, defined daily dose; CV, coefficient of variation.

As shown in Figure 5, among alternative drugs, the price distribution of most therapeutic categories was consistent in three observation periods, indicating the little policy impact on products’ price distribution. In several categories, i.e., C08/09 (Figure 5A), N03 (Figure 5C), N05 (Figure 5D), A07 (Figure 5I), B01 (Figure 5J), and M01 (Figure 5K), the CV of price distribution in alternative drugs were extremely high and exceeded 100%.

### 4.4 Change of Price Ratio

#### 4.4.1 Overall Distribution

**FIGURE 6:**
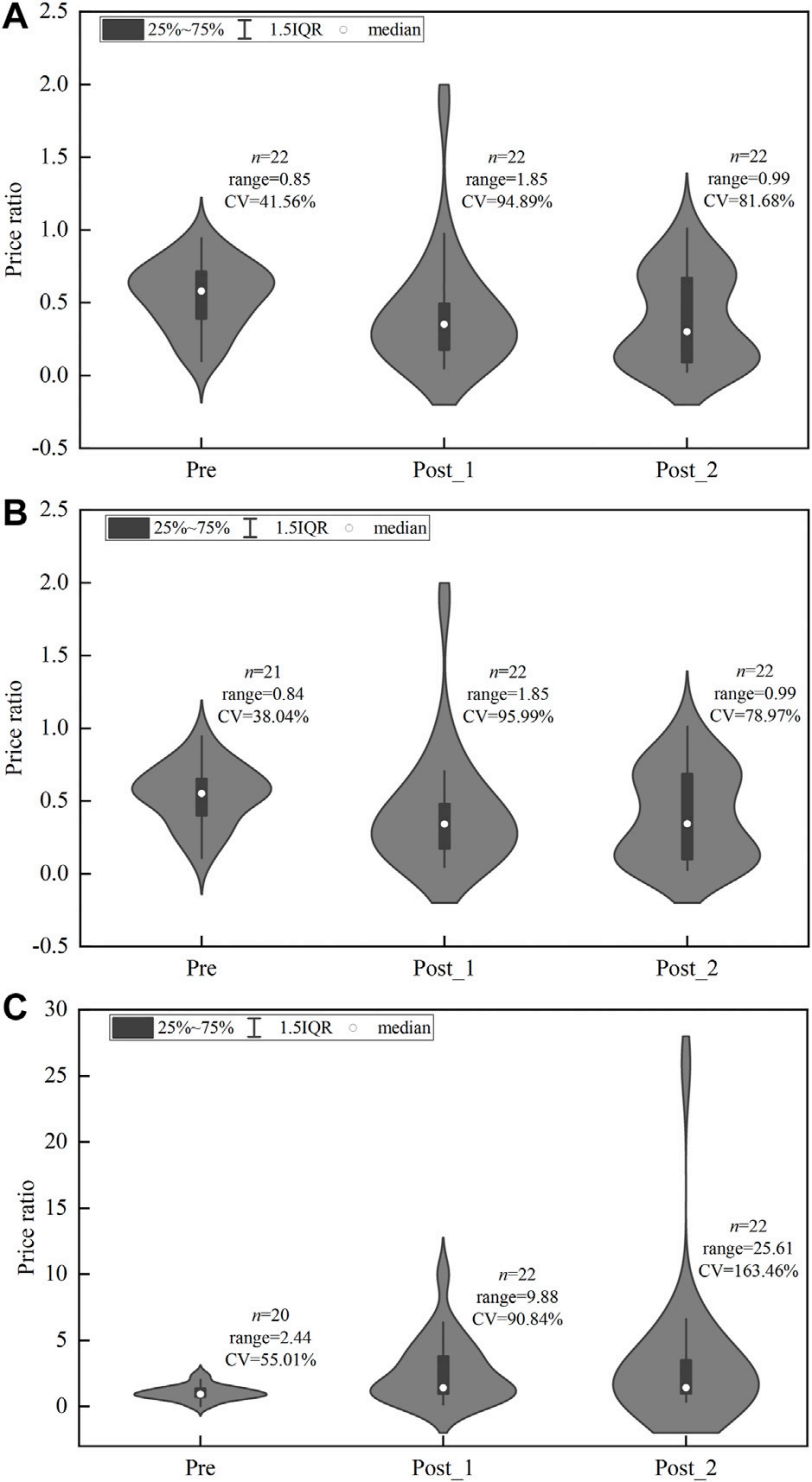
Price ratio distribution of centralized procured INNs among three observation periods. **(A)** PR between generic drugs and original drugs, **(B)** PR between certificated generic drugs and original drugs, **(C)** PR between uncertificated and certificated generic drugs. *Note*: CV, coefficient of variation.

Figure 6 summarizes the distribution of PR among 25 centralized procured INNs in three observation periods. The medians of PR between generic drugs and original drugs were 0.58, 0.35, and 0.30 in the pre-intervention period, first procurement period, and second procurement period respectively (Figure 6A). The medians of PR between certificated generic drugs and original drugs were 0.55, 0.34, and 0.34 in three periods (Figure 6B). The medians of PR between uncertificated and certificated generic drugs were 0.93, 1.42, and 1.42 in three periods. The range (2.44, 9.88, and 25.61) and CV (55.01, 90.84, and 163.46%) of PR between uncertificated and certificated generic drugs showed a prominent upward trend in three periods.

#### 4.4.2 Price Ratio by INN Categories

**FIGURE 7:**
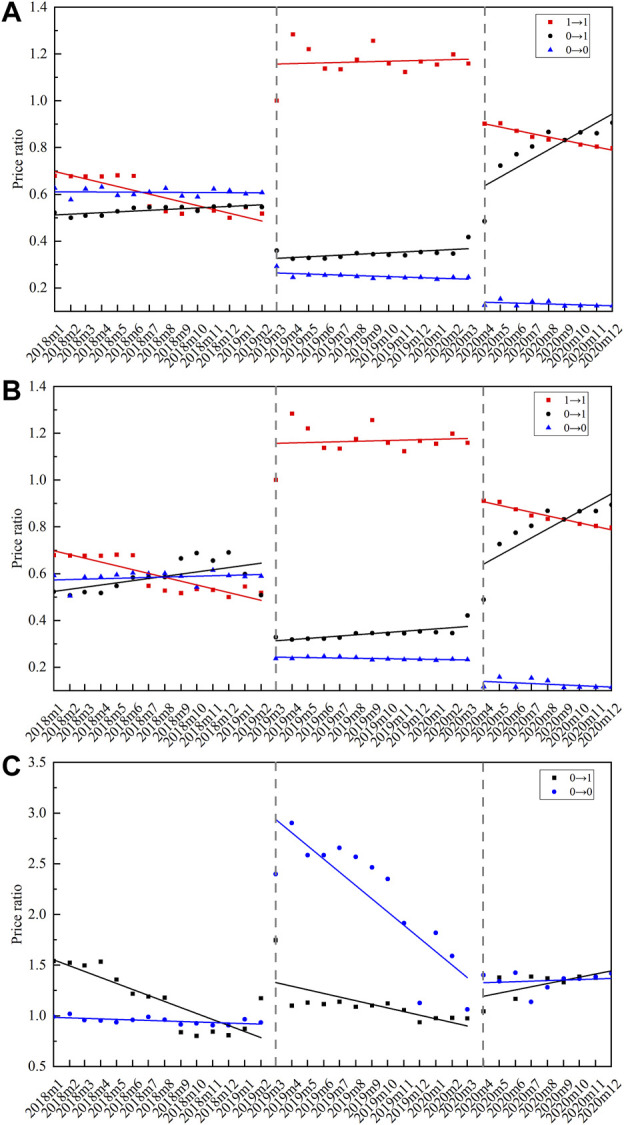
Monthly trends of price ratio among centralized procured INNs with different bid-winning types. **(A)** Price ratio between generic drugs and original drugs, **(B)** price ratio between certificated generic drugs and original drugs, **(C)** price ratio between uncertificated and certificated generic drugs. *Note*: 1→1 represents INNs that the original drug won the bid in two centralized biddings (*n* = 3), 0→1 represents INNs that the original drug only won the bid in the second bidding (*n* = 3), 0→0 represents INNs that original drug did not win the bid in two biddings (*n* = 19).

According to the bid-winning results (including original drug or not) in two centralized biddings, the 25 centralized procured INNs were divided into three groups: 1) INNs that original drug won the bid in two centralized biddings, coded as 1→1; 2) INNs that original drug only won the bid in the second bidding, coded as 0→1; and 3) INNs that original drug did not win the bid in two biddings, coded as 0→0. Figure 7 graphs the monthly change of PR in three INN categories from January 2018 to December 2020. The corresponding ITS results are summarized in Table 3.

**TABLE 3:** Multi-intervention ITS quantifying the impact of centralized procurement policy on price ratio of centralized procured INNs with different bid-winning types.

Categories^a^	Level_1		Trend_1		Level_2		Trend_2	
	*β*_*1*_	*p*	*β*_*2*_	*p*	*β*_*3*_	*p*	*β*_*4*_	*p*
PR between generics and original drugs								
1→1	0.85	**0.000**	0.03	**0.000**	−0.25	**0.000**	−0.02	**0.002**
0→1	−0.54	**0.000**	0.003	0.564	0.50	**0.000**	0.04	**0.031**
0→0	−0.82	**0.000**	−0.01	0.072	−0.52	**0.000**	−0.01	0.555
Overall	−0.50	**0.000**	0.001	0.799	−0.19	**0.000**	0.003	0.483
PR between certificated generics and original drugs								
1→1	0.85	**0.000**	0.03	**0.000**	−0.24	**0.000**	−0.02	**0.001**
0→1	−0.74	**0.000**	−0.002	0.811	0.48	**0.000**	0.04	0.062
0→0	−−0.89	**0.000**	−0.01	**0.049**	−0.48	**0.000**	−0.02	0.183
Overall	−0.60	**0.000**	0.01	**0.002**	−0.06	0.247	−0.03	**0.000**
PR between uncertificated and certificated generics								
1→1	n/a	n/a	n/a	n/a	n/a	n/a	n/a	n/a
0→1	0.49	**0.001**	0.02	0.284	0.24	**0.009**	0.06	**0.001**
0→0	1.30	**0.000**	−0.06	**0.000**	−0.06	0.641	0.07	**0.000**
Overall	0.96	**0.000**	−0.06	**0.000**	0.18	**0.025**	0.08	**0.000**

a 1→1 represents INNs that original drug won the bid in two centralized biddings (*n* = 3), 0→1 represents INNs that original drug only won the bid in the second bidding (*n* = 3), 0→0 represents INNs that original drug did not win the bid in two biddings (*n* = 19).

PR, price ratio. Bold values indicate regression coefficients with statistical significance (*p* < 0.05).

As for the PR between certificated generic drugs and original drugs, the 1→1 category showed an immediate increase (*β* = 0.85, *p* < 0.001) in the first procurement period, and an immediate decline (*β* = -0.24, *p* < 0.001) in the second procurement period. The 0→1 category showed an immediate decrease (*β* = -0.74, *p* < 0.001) in the first procurement period, and an immediate increase (*β* = 0.48, *p* < 0.001) in the second procurement period. The 0→0 category was observed with a significant PR decline in both the first (*β* = -0.89, *p* < 0.001) and second (*β* = -0.48, *p* < 0.001) procurement period. During the second procurement period, the PR of 1→1 and 0→1 category was about 0.8, and that of 0→0 was about 0.15 (Figure 7B).

In regards to the PR between uncertificated and certificated generic drugs, the 0→1 category was observed with immediate PR increment at the start of the first (*β* = 0.49, *p* = 0.001) and second (*β* = 0.24, *p* = 0.009) procurement period. A prominent increment was found in the PR of 0→0 category INNs at the start of the first procurement period (*β* = 1.30, *p* < 0.001), while the change trends significantly declined (*β* = -0.06, *p* < 0.001). During the second procurement period, the PR of 0→1 and 0→0 categories were about 1.0–1.5 (Figure 7C).

In each drug category, the monthly trend of PR between generics and original drugs is quite similar to PR between certificated generics and original drugs (Figures 7A,B), which related to the increased use of certificated generics (especially after policy intervention) and thus resulting in the domination of certificated generics in the price level of overall generic drugs.

## 5 Discussion

As the current world’s largest group purchasing organization, the centralized drug procurement organized by the Chinese government aims to gradually establish a market-driven drug price formation mechanism by adapting regular, institutional, state-level centralized procurement activities. We conducted a multi-intervention ITS to systematically explore the impacts of centralized procurement on drug price and price relations, by using drug procurement data of all public medical institutions in the 11 pilot cities (2018–2020). The study findings first revealed the difference between short-term (within 1-year procurement period) and mid- or long-term (1 year later) impacts of the policy on drug price, as well as the difference between policy impacts on centralized procured drugs (direct effect) and alternative drugs (indirect effect). In addition, the significant impact of the changes in drug price relation upon the policy implementation also provides strong empirical evidence for future policymaking.

### 5.1 Significant Price Decline of the Bid-Winning Drugs After Centralized Biddings

This study first observed significant price reductions of the bid-winning products by each bidding period, generally reflecting the direct price-cutting effect associated with the centralized procurement, which echoes other recent research (Wang et al., 2021). Similar findings were reported by Sigulem and Zucchi (2009) in the evaluation of joint procurement of seven university-affiliated hospitals in Brazil, which observed prominent price changes of the same product in different centrally purchased batches (mainly with price drops). Specifically, this study observed that the price reduction in the second bidding period was significantly smaller and more stable compared with that in the first bidding period, which was generally consistent with market nature and the policy aim of “stabilizing drug price” (NHSA, 2021; Sun et al., 2021). In addition, in the second centralized bidding, the prices of a few INNs did undergo a rise trend. Also, significant differences were observed in the decrease of bid-winning prices among different INNs. Several factors might influence bidding price, findings derived from cross-country studies reported that more potential competitors, decentralized market, and older generation drugs were generally associated with lower centralized bidding prices (Dubois et al., 2021; Wang and Zahur, 2022). Evidence from China indicated that originators are likely to gain lower prices than generics in centralized bidding (Li et al., 2021; Sun et al., 2022). These to some extent explained the difference in price changes among centralized procured INNs in this study. We believe it is necessary for future centralized drug procurement practice to further differentiate and standardize pricing strategies for reshaped groups of varieties and pharmaceutical companies with different features, dynamically improving the sustainability and equity of the policy implementation.

### 5.2 Marked Differences Between Short- and Long-Term Effects in the Price Changes of Non-winning Products

When focusing on non-winning drugs, the study observed that the short-term effect of the price change was stronger than the mid- or long-term effect. In the first bidding period, we observed a drastic decline not only in the price level of bid-winning drugs but also of several non-winning drugs (N→Y products), indicating an emergence of the “spillover effect” brought by the policy implementation. This phenomenon was also reported by other scholars recently (Chen et al., 2020). Wang et al. (2022) and Xie et al. (2021) revealed that the price cut of overall non-winning drugs was mainly attributed to the proactive price reduction of certain original enterprises. However, we found the price of those drugs that failed to win both tenders (we marked N→N category) did not significantly change. Luo et al. (2022) in their latest research also suggested that in the 18-months centralized procurement execution period, the level of price-cut among non-winning medicines (1.54%) was far lower than that of bid-winning counterparts (73.82%). Such evidence indicates that the policy effect on drug price reduction has not achieved universal coverage for all the participating products in the market, in that not all non-winning enterprises showed a willingness to actively lower their product prices.

In the second bidding period observation, the policy effect on the price level and slope of the non-winning products manifested as positive, yet no statistical difference was detected. The price of a subgroup of non-winning products which once won the bid in previous bidding periods (marked as Y→N) showed a significantly increasing trend (14.80%), indicating that these companies might have raised the prices after their products failed to win the bid. Similar changes were mentioned by Pérez et al. (2019) in the evaluation of Colombia’s centralized procurement policy that enterprises intended to rapidly raise their product prices 1 or 2 years after initial price reduction, suggesting a need to assess the long-term effects of such policies on drug prices. In the evaluation of group purchasing on drug prices in French hospitals, Toulemon (2018) observed a slight rise in the price of oligopoly medicines a few months after policy implementation. Therefore, the long-term price-raising inkling observed in this study should be cautiously considered by policymakers. While price fluctuations are common in a market, frequent drug price adjustments, especially those with upward tendencies, are not likely to be the facilitators for achieving stable rational drug use and universal access to healthcare.

### 5.3 Increasingly Scattered Drug Price Distribution and Imbalanced Price Ratio

First, this study found that those drugs which lost the bid throughout the entire course maintained a relatively high price level, with no significant change in their price level and trends. This may indicate that China has not achieved the NHSA’s goal of “uniform between medical insurance payment standards and procurement prices” (NHSA, 2019b) in “4 + 7” pilot cities. These continuous non-winning drugs may mainly come from enterprises with a small market share or with limited competitiveness or capability (e.g., uncertificated generic drugs). Moreover, our analysis of price ratios also found a significantly high price ratio between uncertificated and certificated generics, accompanied by a surge peaking at 2.0–3.0 after the policy execution. Luo et al. (2022) observed a similar rapid increase in the uncertificated generic drug prices (83.18%) in the 18th month of the policy initiation. Such continuously inflated drug prices and deviated price ratios may not only break the rational market equilibrium and affect competition fairness but also stand against the development process of value-based healthcare. These findings indicate that in the context of market-driven centralized procurement, it is of high necessity for proactive policy intervention or engagement in drug-price governance. Efforts should be specifically made on formulating standardized regulation for uncertificated generics’ prices, so as to facilitate the process toward dynamically coordinated drug-pricing practice.

Second, this study observed a further deviated difference between the generic and original drugs in the majority of centralized procured INNs. Before the policy implementation, the price ratio of certificated generics to their original counterparts was around 0.6, slightly higher than the figure in the 2014 Shanghai survey (0.54) (Zhang et al., 2016). While after the policy implementation, the changing level in the price ratio was strongly associated with whether the original product won the bid. The price ratio of INNs with the originator winning the bid was as high as 0.8 after policy intervention. The price of generics reached 70–90% of corresponding originators in the United States, Canada, Japan, and South Korea (Zhang et al., 2016). Whereas in the Netherlands, through insurance companies’ bidding and enhancing prescribing competition, the price of omeprazole and simvastatin generics even down to 2% of pre-patent loss prices (Woerkom et al., 2012). We also found that the price ratio of INNs without an originator winning bid has witnessed a drastic drop to value at 0.2. This widening price difference could be associated with the fact that certain varieties failed to effectively participate in market competition to facilitate a fully competitive market between the originals and generics. Future efforts could be made on increasing the level of diversity and competitiveness in seller market by further incentivizing original enterprises to facilitate their participation in the centralized procurement activities.

Third, the downwardly distributed price intervals of the centrally procured varieties and diminished median prices among most selected pharmaceutical categories in this study could mainly the result of the price cuts of the bid-winning products. However, the variation coefficients of price distribution among most pharmaceutical categories did not significantly decrease, with even remark rising trends in certain therapeutic categories. This may indicate a great potential for future drug price governance to further narrow the price differences among different INNs which share similar clinical categories.

### 5.4 “Spillover Effects” on the Price of Alternative Drugs Upon Policy Implementation

This study has sub-grouped the included drugs by therapeutic categories to explore the policy effect on alternative drugs’ price, while inconsistent patterns were observed in the change of alternative drug price distributions upon policy implementation.

On one hand, from a perspective of short-term policy effect, this study found that the price-cutting effect brought by the centralized procurement policy might at least partially “spillover” to the price change patterns of their alternative counterparts, given the significant decline in the slope of several alternative drugs’ price in the first procurement period. The figure is generally in accordance with the findings reported by Wang et al. (2021) and Hao et al. (2020).

On the other hand, from a dynamic mid-to long-term policy effect perspective, a statistically significant increase (7.04%) in the alternative drug prices was observed since the second bidding period, despite that only a limited number of therapeutic categories experienced such price inclination. It can be explained by the typical “gourd effect” (Yu, 2017) in the course of drug pricing reform, meaning that the magnitude of the price-cutting effect could be neutralized by the increase of unaffected drug prices or other costs. Yu (2020) and Yang et al. (2021) have raised similar concerns about the factual effects on drug prices associated with the centralized procurement policy implementation. Therefore, it would be necessary for more stringent monitoring and regulating measures during the implementation of the centralized procurement policy in a long run; meanwhile, we call for further expanding the drug scope covered by the centralized procurement policy while integrating the categories sharing similar clinical use or functions.

Overall, the inconsistent changing pattern in the price distribution of alternative drugs upon the implementation of the centralized procurement policy in China indicates a limited policy effect on the prices of unaffected INNs. This in turn also presents a deviated trend in the price difference between the bid-winning products and their alternative counterparts. In this situation, to avoid potential risks of market instability and irrational drug use, multiple and coordinated approaches, such as the practice in Scotland (Macbride-Stewart et al., 2021), might be considered pragmatic and effective. Also, in addition to further accelerating the scope of medicines covered by the centralized procurement, measures should be taken to enhance competition at the therapeutic class level rather than simply the INN level.

Several potential limitations should be mentioned regarding the present study. First, this study selected the first batch of “4 + 7” pilot cities for investigating the variations in drug prices over the two-year-long implementation period. Although this facilitated tracking the policy efficacy, the procurement regulations in the “4 + 7” pilot were not sufficiently mature compared to the subsequent batches of centralized procurement. Given the particularities of the “4 + 7” centralized procurement pilot, therefore, extrapolating results of this study to other policy batches should be cautiously performed. Second, since the “4 + 7” pilot was mainly carried out in public medical institutions, this study obtained data for price analysis from the CDSIP database, which extensively covers drug procurement data from public medical institutions. It should be noted that the drug procurement information of private departments (private hospitals, retail pharmacies, etc.), which accounted for about 20% of the total drug consumption, is not covered in the CDSIP database. Therefore, the findings derived from this study mainly present changes in drug prices in public healthcare institutions in China, which may not be fully extended to private departments until further research is conducted.

## 6 Conclusion

The centralized drug procurement policy in China has effectively reduced the price of bid-winning products. In the short term, partial enterprises (mainly originators’ enterprises) that have failed to win the bids have proactively reduced drug prices, accompanied by subsequently dropped prices of alternative drugs under certain therapeutic categories, yielding a coordinated and integrated interaction in the pricing between different categories. Nonetheless, in the long run, the price-cutting effect was likely to be progressively diminished or even largely reversed. Overall, there are several prominent phenomena following the centralized procurement, including but not limited to imbalanced drug price ratio, deviated drug price distribution, abnormally low level of difference between the price of generic and original drugs, and the large price difference between the uncertificated and certificated generics.

Possible policy improvements in the future include: 1) Consistent monitoring and evaluation should be conducted against abnormal price rebounds. 2) Coverage of the centralized procurement list should be expanded based on the therapeutic categories of the medicine. 3) Initiative should be taken to stimulate the original drug enterprises to proactively participate in centralized procurement activities, and to enhance supply-side market competitiveness. 4) Efforts should be made to implement the standard drug medical insurance payment policy promptly.

## Data Availability

The original contributions presented in the study are included in the article/Supplementary Materials, and further inquiries can be directed to the corresponding authors.
