# Supplementary material for: Changing Characteristics of Pharmaceutical Prices in China Under Centralized Procurement Policy: A Multi-Intervention Interrupted Time Series
Source: Front Pharmacol. 2022 Jul 15;13:944540. doi: 10.3389/fphar.2022.944540 (PMC9335887; doi:10.3389/fphar.2022.944540)
Supplement: Supplementary file 1 [file Table1.DOCX]

Supplementary material

**Table S1.** Bid-winning enterprises of each INN in two centralized bidding.

| No. | Centralized  procured INNs | First centralized bidding | Second centralized bidding |
| --- | --- | --- | --- |
| 1 | Amlodipine | Jingxin Pharmaceutical Co.,Ltd. | Jingxin Pharmaceutical Co.,Ltd. Shanghai Fosun High Technology(Group)Co.,Ltd. |
| 2 | Losartan | Hua Hai Pharmaceutical Co.,Ltd. | Hua Hai Pharmaceutical Co.,Ltd. |
| 3 | Irbesartan | Hua Hai Pharmaceutical Co.,Ltd. | Hua Hai Pharmaceutical Co.,Ltd. Hengrui Pharmaceuticals Co., Ltd. |
| 4 | Irbesartan and Hydrochlorothiazide | Hua Hai Pharmaceutical Co.,Ltd. | Hua Hai Pharmaceutical Co.,Ltd. Sanofi Pharmaceuticals Co.,Ltd.* |
| 5 | Fosinopril | Squibb Pharmaceuticals Ltd.* | Hua Hai Pharmaceutical Co.,Ltd. Squibb Pharmaceuticals Ltd.* |
| 6 | Lisinopril | Hua Hai Pharmaceutical Co.,Ltd. | Hua Hai Pharmaceutical Co.,Ltd. |
| 7 | Enalapril | Yangtze River Pharmaceutical Group Co.,Ltd. | Yangtze River Pharmaceutical Group Co.,Ltd. |
| 8 | Atorvastatin | Jialin Pharmaceutical Co.,Ltd. | Lepu Pharmaceutical Co.,Ltd. Qilu Pharmaceutical Co.,Ltd. Xingan Pharmaceutical Co.,Ltd. |
| 9 | Rosuvastatin | Jingxin Pharmaceutical Co.,Ltd. | Jingxin Pharmaceutical Co.,Ltd. |
| 10 | Levetiracetam | Jingxin Pharmaceutical Co.,Ltd. | Jingxin Pharmaceutical Co.,Ltd. |
| 11 | Olanzapine | Hansoh Pharmaceutical Co.,Ltd. | Qilu Pharmaceutical Co.,Ltd Hansoh Pharmaceutical Co.,Ltd. |
| 12 | Risperidone | Hua Hai Pharmaceutical Co.,Ltd. | Hua Hai Pharmaceutical Co.,Ltd. Qilu Pharmaceutical Co.,Ltd |
| 13 | Dexmedetomidine | Yangtze River Pharmaceutical Group Co.,Ltd. | Yangtze River Pharmaceutical Group Co.,Ltd. |
| 14 | Escitalopram | Kelun Pharmaceutical Co.,Ltd. | Chuanning Biotechnology Co.,Ltd. Shanghai Fosun High Technology(Group)Co.,Ltd. |
| 15 | Paroxetine | Hua Hai Pharmaceutical Co.,Ltd. | Hua Hai Pharmaceutical Co.,Ltd. NHU Holding Group Co.,Ltd. |
| 16 | Gefitinib | Astrazeneca Pharmaceutical Co.,Ltd.* | Astrazeneca Pharmaceutical Co.,Ltd.* Qilu Pharmaceutical Co.,Ltd Zhengda Tianqing Pharmaceutical Co.,Ltd. |
| 17 | Imatinib | Hansoh Pharmaceutical Co.,Ltd. | Zhengda Tianqing Pharmaceutical Co.,Ltd. Hansoh Pharmaceutical Co.,Ltd. |
| 18 | Pemetrexed | Huiyu Pharmaceutical Co.,Ltd. | Lilly Pharmaceutical Co.,Ltd.* Huiyu Pharmaceutical Co.,Ltd. |
| 19 | Cefuroxime | Brilliant Pharmaceutical Co., Ltd. | Jingxin Pharmaceutical Co.,Ltd. Sinopharm Zhijun(Shenzhen)Pharmaceutical Co.,Ltd. Brilliant Pharmaceutical Co., Ltd. |
| 20 | Entecavir | Zhengda Tianqing Pharmaceutical Co.,Ltd. | Dawnrays Pharmaceutical Co.,Ltd. Fujian Aohua Group Co., Ltd. |
| 21 | Tenofovir Disoproxil | Brilliant Pharmaceutical Co., Ltd. | Brilliant Pharmaceutical Co., Ltd. Qilu Pharmaceutical Co.,Ltd |
| 22 | Montmorilonite | Simcere Pharmaceutical Co.,Ltd. | Harbin Pharmaceutical Group Holding Co.,Ltd. Warrant Pharmaceutical Co.,Ltd Hailisheng Pharmaceutical Co., Ltd. |
| 23 | Clopidogrel | Salubris Pharmaceuticals Co.,Ltd. | Sanofi Pharmaceuticals Co.,Ltd.* Salubris Pharmaceuticals Co.,Ltd. |
| 24 | Flurbiprofen | Tide Pharmaceutical Co.,Ltd.* | Tide Pharmaceutical Co.,Ltd.* |
| 25 | Montelukast | Anbisheng Pharmaceutical Technology Co. Ltd | Anbisheng Pharmaceutical Technology Co. Ltd |

*Note*: * enterprise of original drugs. INN, International Nonproprietary Name.

**Table S2.** The name of drugs included in this study.

| No. | Centralized  procured drugs | ATC code | Alternative drugs |
| --- | --- | --- | --- |
| 1 | Amlodipine | C08CA01 | Levoamlodipine, Amlodipine folate, Felodipine, Nifedipine, Amlodipine and Atorvastatin, Amlodipine and Benazepril, Olmesartan and Amlodipine, Valsartan and Amlodipine |
| 2 | Losartan | C09CA01 | Candesartan, Valsartan, Telmisartan, Olmesartan, Allisartan, Olmesartan and Amlodipine, Valsartan and Amlodipine, Olmesartan and Hydrochlorothiazide, Losartan and Hydrochlorothiazide, Telmisartan and Hydrochlorothiazide, Valsartan and Hydrochlorothiazide, Candesartan and Hydrochlorothiazide |
| 3 | Irbesartan | C09CA04 | Olmesartan, Candesartan, Valsartan, Telmisartan, Losartan, Allisartan, Olmesartan and Amlodipine, Valsartan and Amlodipine, Olmesartan and Hydrochlorothiazide, Losartan and Hydrochlorothiazide, Telmisartan and Hydrochlorothiazide, Valsartan and Hydrochlorothiazide, Irbesartan and Hydrochlorothiazide, Candesartan and Hydrochlorothiazide |
| 4 | Irbesartan and Hydrochlorothiazide | C09DA04 | Olmesartan and Hydrochlorothiazide, Losartan and Hydrochlorothiazide, Telmisartan and Hydrochlorothiazide, Valsartan and Hydrochlorothiazide, Candesartan and Hydrochlorothiazide, Olmesartan, Candesartan, Valsartan, Telmisartan, Allisartan, Olmesartan and Amlodipine, Valsartan and Amlodipine |
| 5 | Fosinopril | C09AA09 | Captopril, Benazepril, Perindopril, Ramipril, Amlodipine and Benazepril, Benazepril and Hydrochlorothiazide, Compound Captopril, Lisinopril and Hydrochlorothiazide, Enalapril folate, Perindopril and Indapamide, Perindopril and Amlodipine |
| 6 | Lisinopril | C09AA03 | Captopril, Benazepril, Enalapril, Perindopril, Ramipril, Amlodipine and Benazepril, Benazepril and Hydrochlorothiazide, Compound Captopril, Lisinopril and Hydrochlorothiazide, Enalapril folate, Perindopril and Indapamide, Perindopril and Amlodipine |
| 7 | Enalapril | C09AA02 | Captopril, Benazepril, Perindopril, Ramipril, Amlodipine and Benazepril, Benazepril and Hydrochlorothiazide, Compound Captopril, Lisinopril and Hydrochlorothiazide, Enalapril folate, Perindopril and Indapamide, Perindopril and Amlodipine |
| 8 | Atorvastatin | C10AA05 | Pitavastatin, Pravastatin, Fluvastatin, Simvastatin, Lovastatin, Ezetimibe and Simvastatin, Xuezhikan, Zhibituo, Zhibitai |
| 9 | Rosuvastatin | C10AA07 | Pitavastatin, Pravastatin, Fluvastatin, Simvastatin, Lovastatin, Ezetimibe and Simvastatin, Xuezhikan, Zhibituo, Zhibitai |
| 10 | Levetiracetam | N03AX14 | Oxcarbazepine, Magnesium Valproate, Valproate Sodium, Carbamazepine, Lamotrigine, Topiramate |
| 11 | Olanzapine | N05AH03 | Paliperidone, Clozapine, Aripirazole, Metamizole and Chlorpromazine, Amisulpride, Loxapine, Haloperidol, Quetiapine, Fluphenazine Decanoate, Chlorpromazine, Ziprasidone, Trifluoperazine, Ziprasidone |
| 12 | Risperidone | N05AX08 | Quetiapine, Aripirazole, Metamizole and Chlorpromazine, Amisulpride, Loxapine, Haloperidol, Quetiapine, Fluphenazine Decanoate, Chlorpromazine, Ziprasidone, Trifluoperazine, Ziprasidone, Perphenazine, Sulpiride, Penfluridol, Piperothiazide, Piperothiazide, Tiapride |
| 13 | Dexmedetomidine | N05CM18 | Midazolam, Diclofenac |
| 14 | Escitalopram | N06AB10 | Citalopram, Fluoxetine, Paroxetine, Votioxetine, Duloxetine, Sertraline, Fluvoxamine, Bupropion, Trazodone, Venlafaxine |
| 15 | Paroxetine | N06AB05 | Fluoxetine, Votioxetine, Duloxetine, Escitalopram, Citalopram, Sertraline, Fluvoxamine, Bupropion, Trazodone, Venlafaxine |
| 16 | Gefitinib | L01EB01 | Icotinib, Erlotinib, Afatinib, Osimertinib |
| 17 | Imatinib | L01XE01 | Nilotinib, Dasatinib |
| 18 | Pemetrexed | L01BA04 | - |
| 19 | Cefuroxime | J01DC02 | Cefaclor, Cefalexin, Cefprozil, Cefdinir, Cefmnoxime, Cefixime, Cefadroxil |
| 20 | Entecavir | J05AF10 | Tenofovir Disoproxil, Tenofovir Alafenamide, Lamivudine, Adefovir, Telbivudine |
| 21 | Tenofovir Disoproxil | J05AF07 | Tenofovir Alafenamide, Lamivudine, Adefovir, Telbivudine |
| 22 | Montmorilonite | A07BC05 | Berberine, Loperamide, Albumini Tannas |
| 23 | Clopidogrel | B01AC04 | Ticagrelor, Aspirin, Tiolopidine, Cilostazol |
| 24 | Flurbiprofen | M01AE09 | Parecoxib, Piroxicam, Diclofenac, Tromethamine, Indomethacin |
| 25 | Montelukast | R03DC03 | Pemirolast , Pranlukast, Seratrodast, Ibudilast, Zafirlukast |

Note: ATC, Anatomical Therapeutic and Chemical.
